# The alteration of the structure and macroscopic mechanical response of porcine patellar tendon by elastase digestion

**DOI:** 10.3389/fbioe.2024.1374352

**Published:** 2024-04-17

**Authors:** Xiaoyun Liu, Yuping Deng, Zeyu Liang, Dan Qiao, Wentian Zhang, Mian Wang, Feifei Li, Jiannan Liu, Yaobing Wu, Guangxin Chen, Yan Liu, Wenchang Tan, Jian Xing, Wenhua Huang, Dongliang Zhao, Yanbing Li

**Affiliations:** ^1^ National Key Discipline of Human Anatomy, Guangdong Provincial Key Laboratory of Medical Biomechanics, Guangdong Engineering Research Center for Translation of Medical 3D Printing Application, School of Basic Medical Sciences, Southern Medical University, Guangzhou, China; ^2^ Institute of Biomedical Engineering, Shenzhen Bay Laboratory, Peking University Shenzhen Graduate School, Shenzhen, China; ^3^ Department of Orthopedics and Traumatology, Integrated Hospital of Traditional Chinese Medicine, Southern Medical University, Guangzhou, China; ^4^ Department of Pathology, Sir Run Run Shaw Hospital, Zhejiang University, Hangzhou, Zhejiang, China; ^5^ The School of Basic Medical Sciences, Fujian Medical University, Fujian, China; ^6^ Department of Orthopaedics, Pingshan General Hospital of Southern Medical University, Shenzhen, China; ^7^ Medical Image College, Mudanjiang Medical University, Mudanjiang, Heilongjiang, China; ^8^ Department of Anatomy, Gannan Healthcare Vocational College, Ganzhou, China; ^9^ Guangdong Medical Innovation Platform for Translation of 3D Printing Application, The Third Affiliated Hospital of Southern Medical University, Guangzhou, China

**Keywords:** patellar tendon, elastin, nonlinearity, stress relaxation, two-photon imaging

## Abstract

**Background:** The treatment of patellar tendon injury has always been an unsolved problem, and mechanical characterization is very important for its repair and reconstruction. Elastin is a contributor to mechanics, but it is not clear how it affects the elasticity, viscoelastic properties, and structure of patellar tendon.

**Methods:** The patellar tendons from six fresh adult experimental pigs were used in this study and they were made into 77 samples. The patellar tendon was specifically degraded by elastase, and the regional mechanical response and structural changes were investigated by: (1) Based on the previous study of elastase treatment conditions, the biochemical quantification of collagen, glycosaminoglycan and total protein was carried out; (2) The patellar tendon was divided into the proximal, central, and distal regions, and then the axial tensile test and stress relaxation test were performed before and after phosphate-buffered saline (PBS) or elastase treatment; (3) The dynamic constitutive model was established by the obtained mechanical data; (4) The structural relationship between elastin and collagen fibers was analyzed by two-photon microscopy and histology.

**Results:** There was no statistical difference in mechanics between patellar tendon regions. Compared with those before elastase treatment, the low tensile modulus decreased by 75%–80%, the high tensile modulus decreased by 38%–47%, and the transition strain was prolonged after treatment. For viscoelastic behavior, the stress relaxation increased, the initial slope increased by 55%, the saturation slope increased by 44%, and the transition time increased by 25% after enzyme treatment. Elastin degradation made the collagen fibers of patellar tendon become disordered and looser, and the fiber wavelength increased significantly.

**Conclusion:** The results of this study show that elastin plays an important role in the mechanical properties and fiber structure stability of patellar tendon, which supplements the structure-function relationship information of patellar tendon. The established constitutive model is of great significance to the prediction, repair and replacement of patellar tendon injury. In addition, human patellar tendon has a higher elastin content, so the results of this study can provide supporting information on the natural properties of tendon elastin degradation and guide the development of artificial patellar tendon biomaterials.

## 1 Introduction

Patellar tendon injury is usually caused by repeated high loads, which often happens to athletes who need to repeat the maximal jumps (basketball or volleyball) (Reinking, 2016). As many as 50% excellent basketball and volleyball players suffer from patellar tendon injury (Burton, 2022). However, the operation or repair strategy for the patellar tendon has a high re-injury or rupture rate, limiting their efficiency in restoring the original structure and function of the patellar tendon. Tissue engineering of the tendons is considered a novel strategy for repair and regeneration. The structure-function relationship of tissues remains a key goal in tissue engineering (Wang et al., 2018). Biomaterials must have mechanical properties similar to those of the natural tissue structure, vital in stress transmission and load-bearing in the early stage of patellar tendon regeneration (Zhao et al., 2020; Russo et al., 2022). Therefore, an in-depth understanding of the microstructure and biomechanical properties of the patellar tendon tissue aid in understanding the pathogenesis of injury, developing surgical and reconstructive effects.

Research has shown that a few proteins or non-protein components combine in complex tissue structures, affecting the nonlinear and stress relaxation behaviors of materials (Zitnay and Weiss, 2018). The diversity of tendon components and their complex structures, such as collagen fibers, elastic fibers, and glycosaminoglycans (GAGs), are crucial for the normal mechanical function of the tendon. Clinical research has shown that elastic fibers are affected by hereditary diseases such as Marfan syndrome and skin laxity, causing joint relaxation which damages the mechanical integrity of the tendons and connective tissues near the joints (Zhang et al., 2022; Krarup et al., 2023). Elastic fibers degenerate during aging, possibly causing a loss of anti-fatigue ability and increasing the risk of tendon injury in older adults (Eekhoff et al., 2023). As a dense connective tissue, elastin in the patellar tendon can combine with molecules, such as decorin and dimers, affecting the mechanical properties of the tendon (Beach et al., 2022). Research has shown that elastic fibers provide elasticity that can restore the tissue to its original state after mechanical deformation and that elastin fibers store elastic energy, protecting collagen fibers from impact loads (Fazaeli et al., 2020).

The elastin content and function of different species and types of tendons are different, and the elastin content of human tendons is the highest, compared with pigs, cows and mice (Eekhoff et al., 2023). Before entering the experimental study of human tendon injury repair, it is generally verified on animal models. It is considered that porcine tendon is a qualified substitute for biomechanical research of human tendon repair *in vitro* (Burgio et al., 2023), and porcine patellar tendon is also a medium commonly used in tissue engineering to verify that biological scaffold (Wang et al., 2018) and hydrogel (Freedman et al., 2022a) promote tendon repair and regeneration. Studies show that the elastic protein content of energy storage tendon is more than that of position tendon (Godinho et al., 2017). Patellar tendon, as one of the energy storage tendons, needs greater ductility and elasticity when walking or exercising (Thorpe et al., 2016). The patellar tendon is an anisotropic nonlinear viscoelastic material, and its stress-strain curve exhibits typical nonlinearity (Mohamed et al., 2020). The stress relaxation test exhibited typical time-dependent behavior. Previous studies used elastase to treat pig tricuspid valve anterior lobe, pig thoracic aorta and human upper eyelid, so as to explore the influence of elastin on them (Ugradar et al., 2020; De Moudt et al., 2021; Salinas et al., 2022). Hence, enzymatic degradation is a recent focus to study the relationship between elastin and biomechanics. The correlation between chemical composition and biomechanical properties of bovine patellar tendon shows that elastin content may predict the mechanical properties of patellar tendon, such as Young’s modulus and stiffness (Ristaniemi et al., 2020). However, the mechanical analysis of patellar tendon after specific degradation of elastin has not been studied. Elastin is an essential component affecting the viscoelastic properties of the tissue extracellular matrix and is crucial for tissue ductility (Urbanczyk et al., 2020). Although elastase treatment significantly affects the hysteresis experiment of tissue (Godinho et al., 2021), the existing research has not completely clarified the effect of elastin on the stress relaxation characteristics of viscoelastic tissue. The proximal patellar tendon is considered a common injury site (Pearson and Hussain, 2014), but the influence of elastin on the mechanical properties and structure of its region has not been better explored. Thus, it is necessary to completely investigate elastin’s role in the mechanical response of patellar tendon in axial tension and stress relaxation and its influence on structure.

Therefore, this study aimed to characterize the effects of elastin degradation on the structure, quasi-static tensile material properties, and viscoelastic properties of different porcine patellar tendon regions. The mechanical behavior of patellar tendon under the change of elastin fiber content and distribution was quantitatively analyzed by uniaxial stretching. Furthermore, establishing a dynamic constitutive model of patellar tendon before and after elastin degradation. The numerical calculations can be used to optimize the surgical patellar tendon reconstruction technology and select grafts, and guide the development of biomaterial scaffolds. Histological analysis and two-photon imaging were used to study the effect of elastin degradation on the microstructure of patellar tendon.

## 2 Materials and methods

### 2.1 Sample preparation

The patellar tendons from six fresh adult experimental pigs (Bama Xiao Xiang pig, weighing 35–50 kg, approximately 12-months old, mixed sex) were dissected (Mariano et al., 2023), and the specimens were free from disease and injury, as approved by the Ethics Committee of Shenzhen Bay Laboratory. The left and right patellar tendons were obtained from each pig, and the difference between them were not discussed in this study. Then the patellar tendons were wrapped with phosphate-buffered saline (PBS)-soaked gauze, and stored frozen at −20 °C (Maeda et al., 2021) until needed. Except the damaged samples, 77 samples were used in this study (Supplementary Table S1).

### 2.2 Elastase digestion

Based on previous studies (Liu et al., 2023), the samples were divided into two groups: 5 U/mL elastase, PBS. The two groups both contained 1χPBS and 0.1 mg/mL soybean trypsin inhibitor (SBTI) solution. And the samples in the two group were incubated for 8 h at room temperature. To further confirm the feasibility of this treatment protocol, the samples were washed thrice in PBS for subsequent biochemical analysis.

### 2.3 Biochemical analysis

Collagen, GAGs, and total protein contents were quantified in the patellar tendons incubated with elastase solution or PBS buffer. Collagen was quantified through hydroxyproline detection using a hydroxyproline detection kit (Solarbio, BC0255) according to the manufacturer’s instructions (n = 18, n = 3 for each). Hydroxyproline is the iconic amino acid of collagen, accounting for approximately 13.5% of collagen. The total collagen content is 7.5-fold higher than that of hydroxyproline (Stoilov et al., 2018). The GAGs were quantitatively analyzed according to the instructions of the GAGs Test Kit (Biocolor, B1000). Ninhydrin colorimetry was used to quantitatively analyze the total protein content. Compared to the amino acid standard, the total protein content can be indirectly quantified (Starcher, 2001).

### 2.4 Mechanical test

**FIGURE 1:**
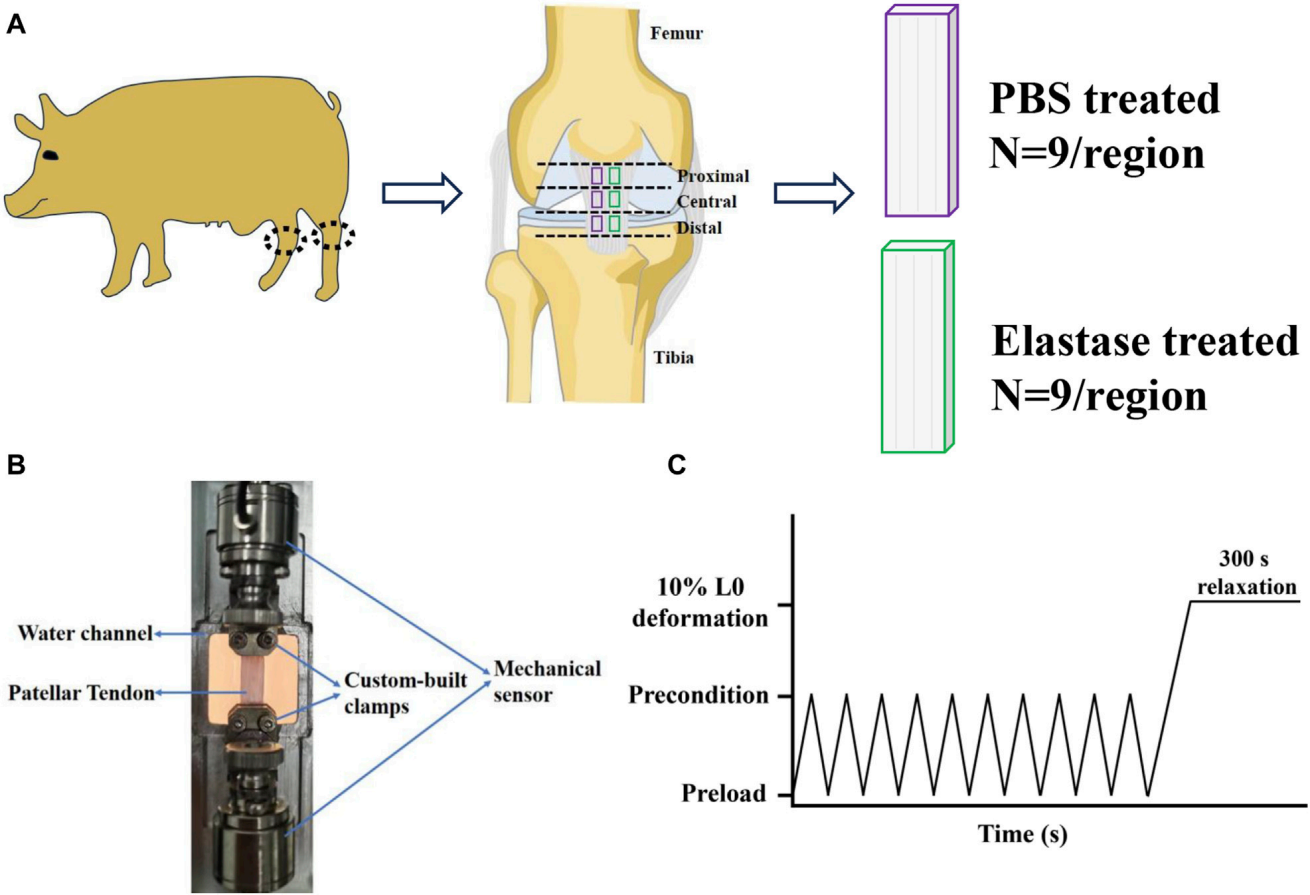
Sample preparation and mechanical test of patellar tendon. **(A)** Division of patellar tendon region and preparation of grouped samples. The black dotted frame is the knee joint. PBS is phosphate-buffered saline. **(B)** Experimental device for uniaxial mechanical testing of the patellar tendon sample. **(C)** Mechanical test protocol.

The patellar tendons were taken out of the refrigerator at −20 °C and placed in 1χPBS buffer for 30–60 min at room temperature to completely thaw (Buján et al., 2000; Lee and Eliott, 2017; Darrieutort-Laffite et al., 2023; Solis-Cordova et al., 2023). Then they were evenly divided into three parts along the direction from the femur to the tibia (n = 9, proximal, central, and distal; Figure 1A (Rigozzi et al., 2009). The thickness of the patellar tendon prepared using cryomicrotome was approximately 550 μm, and the actual thickness of the patellar tendon was measured using a thickness gauge. Subsequently, the patellar tendon was divided into two test samples with dimensions of approximately 4.5χ20 mm (for fixation), and the actual width was measured using a Vernier caliper to calculate the cross-sectional area (Figure 1A). Finally, a 3D printed auxiliary loading device of 10 mm was used to assist in the loading of the test sample.

This study involved repeated mechanical tests; therefore, finding a repeatable mechanical protocol was essential to reduce the influence of the stretching times on the results. The results of repeated mechanical tests showed that the second and third tensile or stress relaxation mechanical data differed insignificantly, indicating that their tests were repetitive (Supplementary Figures S1A–M). Thus, the mechanical data of the second stretch was selected as the mechanical property baseline of the patellar tendon before incubation and used for subsequent mechanical data analysis. The samples were tested by tensile testing machine (CARE Measurement and Control Co. Ltd.), and they were gripped by custom-built clamps (Figure 1B). During the mechanical test, the samples were soaked in 1χPBS solution to keep hydrated. A tensile test and stress-relaxation test were performed according to the protocol (Figure 1C): After the mechanical sensor returned to zero, the sample was preloaded with 0.01 N to remove slack, preconditioned with 10 triangular wave cycles from 0.01 N to 0.02 N at a speed of 0.01 N/s and a frequency of 0.25 Hz to stabilize the sample (Yamamoto et al., 1999; Vafek et al., 2018). Then return to the preloaded 0.01N (0.01N was the preload. When the sample ended 10 cycles, the equipment made it stay at the position of 0.02N, so it needed to return to the preload state, that is, to 0.01N), and record the distance from clamp to clamp at this moment as the initial length L_0_ of the sample (Eskandari et al., 2018). Then, the sample was uniaxial loaded to 10% deformation at the speed of 1%/s (Lee and Eliott, 2017; Smith et al., 2019) to evaluate the elastic characteristics of patellar tendon (Henninger et al., 2015). After reaching 10% deformation, it was kept for 300 s to study the viscoelastic stress relaxation response (Castile et al., 2016; Eskandari et al., 2018). This completed the first mechanical test. After standing for 1 min, the sample was returned to its initial length (recorded in the first test) and the second stretching-relaxation was repeated. Subsequently, the clamps were removed from the mechanical machine, along with the samples. Two test samples from the same region were randomly divided into PBS and elastase group (Figure 1A). They were soaked in 0.1 mg/mL SBTI solution for 15 min. And then the control group was treated with PBS solution (containing 0.1 mg/mL SBTI solution), while the treated group was incubated with 5 U/mL elastase solution, both of which were incubated at room temperature for 8 h. After incubation, the samples were washed three times with PBS. Then the clamps with test samples were placed on a stretching machine. The same initial length recorded in the first mechanical test was loaded, and then the sample was retested with the same mechanical protocol. The mechanical baseline of each group before incubation was used as a control.

### 2.5 Constitutive model

#### 2.5.1 Hyperelastic constitutive model

Based on the mechanical test results of this study, the nonlinear constitutive equation was used to describe the strain energy density function of patellar tendon deformation. The constitutive models commonly used to represent tendons were Yeoh model (Zumbrunn et al., 2018), Ogden model (Bajuri et al., 2016), Fung-model (Ngwangwa et al., 2022), *etc.* As the arrangement of fibers and bundles is approximately unidirectional, tendon is considered as a transversely isotropic material (Böl et al., 2015). In the transversely isotropic constitutive model, Yeoh model has the fastest convergence speed and the most stable performance in all initial parameter estimation (Ngwangwa and Nemavhola, 2021), and it is one of the solutions for rapid mechanical response modeling of tendons (Ekiert et al., 2021). Therefore, assuming that the patellar tendon is incompressible, transversely isotropic, and hyperelastic, Yeoh model was used to establish the material properties of the patellar tendon and simulate their tensile mechanical changes before and after elastin degradation. The strain energy function of Yeoh model can be expressed as (Ngwangwa and Nemavhola, 2021):

$$W=\sum_{i=1}^{N}C_{i0}{\left(I_{1}-3\right)}^{i}$$ (1)

For incompressible materials, the typical parameters were expressed as:

$$W=C_{10}\left(I_{1}-3\right)+C_{20}{\left(I_{1}-3\right)}^{2}+C_{30}{\left(I_{1}-3\right)}^{3}$$ (2)

where W is the strain energy density function, N = 3, I is a nonzero natural number, and I_1_ is an invariant. C_10_, C_20_ and C_30_ are material parameters indicating the stiffness of the material and were obtained by fitting the experimental data.

#### 2.5.2 Viscoelastic constitutive model

Prony series is a model of viscoelastic materials commonly used in engineering, which can be used to better simulate the time-dependent behavior of materials (Bose et al., 2020; Park et al., 2023). Therefore, the viscoelasticity of the patellar tendon was characterized by a second-order Prony series (Shearer, et al., 2020), and the stress-time data from the stress relaxation experiment were analyzed. Young’s modulus E was obtained from the stress relaxation experiment data. Assuming that the patellar tendon was an incompressible material, the Poisson’s ratio was set to a constant, that is, ν = 0.5 (Mihai and Goriely, 2017), and the shear modulus G was calculated. The formula is as follows (Maritz et al., 2021):

$$\sigma =\frac{F}{\text{CSA}}$$ (3)

$$\varepsilon =\frac{∆L}{L_{0}}$$ (4)

$$Ε=\frac{\sigma }{\varepsilon }$$ (5)

$$G=\frac{E}{2\left(1+v\right)}$$ (6)

where σ is stress, ε is strain, F is load, and CSA is cross-sectional area; L_0_ is initial length, and ΔL is length variation.

The formula for the shear modulus of the relaxation effect of the Prony coefficient is as follows (Pan et al., 2022):

$$G\left(t\right)=G_{\infty }+\sum_{i=1}^{N}G_{i}e^{\frac{-t}{\tau_{i}}}=G_{0}\left(\alpha_{\infty }+\sum_{i=1}^{N}\alpha_{i}.e\frac{-t}{\tau_{i}}\right)$$ (7)

$$\alpha_{\infty }=1-\sum_{i=1}^{N}\alpha_{i}$$ (8)

where G_0_ is the instantaneous shear modulus, G(t) is the shear modulus of relaxation effect, t is time, N is the number of Prony series, α_i_ is the material parameter of correlation modulus, and τ_i_ is the material parameter of relaxation time.

### 2.6 Histological analysis

The patellar tendons incubated with PBS or elastase were fixed in 4% paraformaldehyde for 48 h after mechanical testing, and embedded in paraffin after dehydration. Three paraffin longitudinal sections with a thickness of 5 μm were obtained in the middle of the patellar tendon continuously, and Verhoeff’s Van Gieson (VVG) staining was performed (n = 3), exhibiting collagen fibers in red and elastin fibers in black. Standard images representing collagen and elastic fibers were selected for analysis. Six areas were selected in the film, and ImageJ software was used to calculate the wavelength of the collagen fibers. The wavelength was defined as the distance between two consecutive bending peaks. The wavelength of six consecutive peaks was calculated, and the average value was used for statistical analysis.

A fresh experimental porcine patellar tendon was obtained, and the patellar tendon was divided into three regions according to mechanical test standards: proximal, central, and distal. The fixation of patellar tendon was the same as VVG staining. Structural differences among the three patellar tendon regions were identified using Movat’s staining. This staining revealed collagen fibers (yellow), smooth muscle cells (red), proteoglycans or matrix (blue-green), elastic fibers or nuclei (purple to black), and foam cells (purple).

### 2.7 Two-photon microscopy

Two photon microscopy (Olympus, FVMPE-RS) with 40χwater lens (NA 0.8) was used to collect the microstructure of patellar tendon and to analyze the effect of elastin degradation on collagen fiber. A central patellar tendon was made to the dimensions consistent with the mechanical test and divided into two samples, which were randomly divided into PBS group and elastase treatment group (Figure 1A). The clamps for mechanical test were fixed in a 60 mm Petri dish with glue and gripped fresh samples, which were at the same horizontal line without stress. The samples were imaged in pure water, and the collagen fibers were visualized by second harmonic generation (SHG, excitation: 840nm; emission: 410–460 nm) (Eekhoff et al., 2020). Randomly select a field of view. Images were acquired with a resolution of 4096 × 4096 pixels, and sampling speed was 2.0 us/pixel. Once imaging was completed, 12 mL of PBS or 5 U/mL elastase solution was added to the dish, respectively, and the samples were incubated for 8 h at room temperature. *In-situ* second harmonic imaging was performed on the treated sample again. Since it was difficult to find the region consistent with the pre-treated imaging, the imaging region was randomly selected for the treated samples.

### 2.8 Data analysis

**FIGURE 2:**
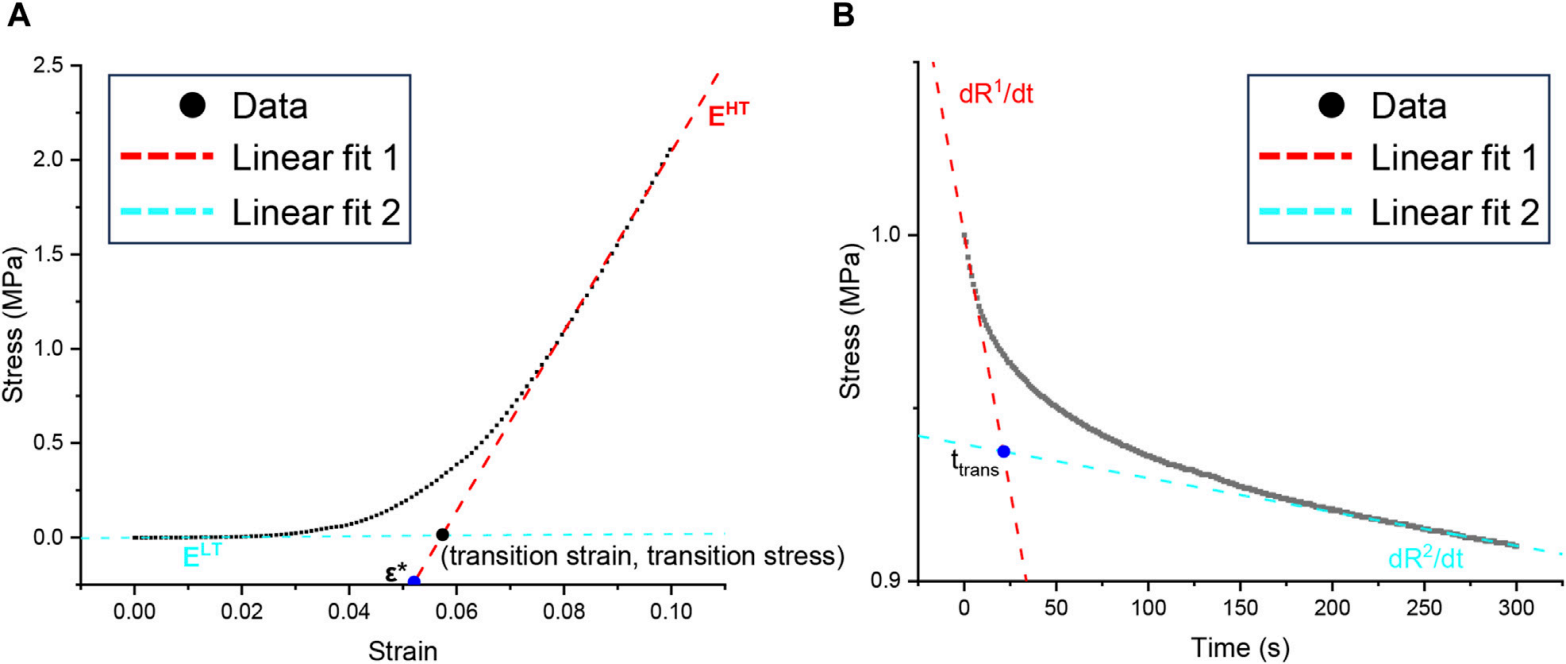
Graphic analysis of mechanical data. **(A)** Parameters quantified from uniaxial tension tests, including the low-tension modulus E^LT^, the high-tension modulus E^HT^, the index of extensibility ε^*^, the transition strain, and the transition stress. **(B)** Parameters quantified from stress relaxation data, including the initial slopes dR^1^/dt, the saturated slopes dR^2^/dt, and the transition time t_trans_.

The tensile stress and strain were calculated using Eqs 3, 4, respectively. The slopes of the low- and high-strain linear regions on the stress-strain curve were obtained by bidirectional curve fitting and were recorded as low (E^LT^) and high (E^HT^) tensile moduli, respectively (Herbert et al., 2016; Song et al., 2022). The intersection of the two tangents defined a transition point, with the abscissa of the transition strain and the ordinate of the transition stress, and the intersection of the tangent of the high-strain linear region and the *X*-axis was recorded as the ductility index ε *. A data analysis diagram is shown in Figure 2A (Pineda-Castillo et al., 2022).

In the stress-relaxation part, the stress was normalized. The peak stress was the maximum stress after stretching to 10% deformation, the equilibrium stress was the steady stress after stress relaxation for 300 s, and the relaxation percentage was the relative change value after relaxation for 300 s. On the stress-time curve, the initial slope dR^1^/dt was the linear fitting of the 5 s data, and the saturation slope dR^2^/dt was the linear fitting of the last 50 data points. The intersection of the two tangents was used as an index of the transition time, which was used to quantitatively evaluate the shape of the stress-relaxation curve. The data analysis diagram is shown in Figure 2B (Duginski et al., 2020).

### 2.9 Statistical analysis

The statistical analysis software GraphPad Prism nine was used to sort and statistically analyze the data. All data were expressed as mean ± standard deviation, and Shapiro–Wilk test was used to test the normality of all the data. If the experimental data conformed to the normal distribution, the basic data and mechanical baseline of the different patellar tendon areas were compared using a single-factor analysis of variance combined with a *post hoc* Bonferroni’s test for multiple comparisons. The remaining biochemical indices and wavelengths of the PBS treatment and elastase incubation groups were analyzed using a two-sample *t*-test. If it did not conform to the normal distribution, the Kruskal–Wallis test was used, and a *post hoc* Dunnett’s test was used for multiple comparisons. According to the normality of the data, a paired *t*-test was used to evaluate the mechanical properties of samples incubated with elastase or PBS before and after treatment; otherwise, the Wilcoxon signed-rank test was used for paired samples. For all analyses, *p < 0.05 was considered significant.

## 3 Results

### 3.1 Baseline data comparison

There was no significant difference in the cross-sectional area and initial length of the mechanical test samples of different groups (Supplementary Figures S2A, B), which indicated that different samples could be used for comparison.

### 3.2 Biochemical results

**FIGURE 3:**
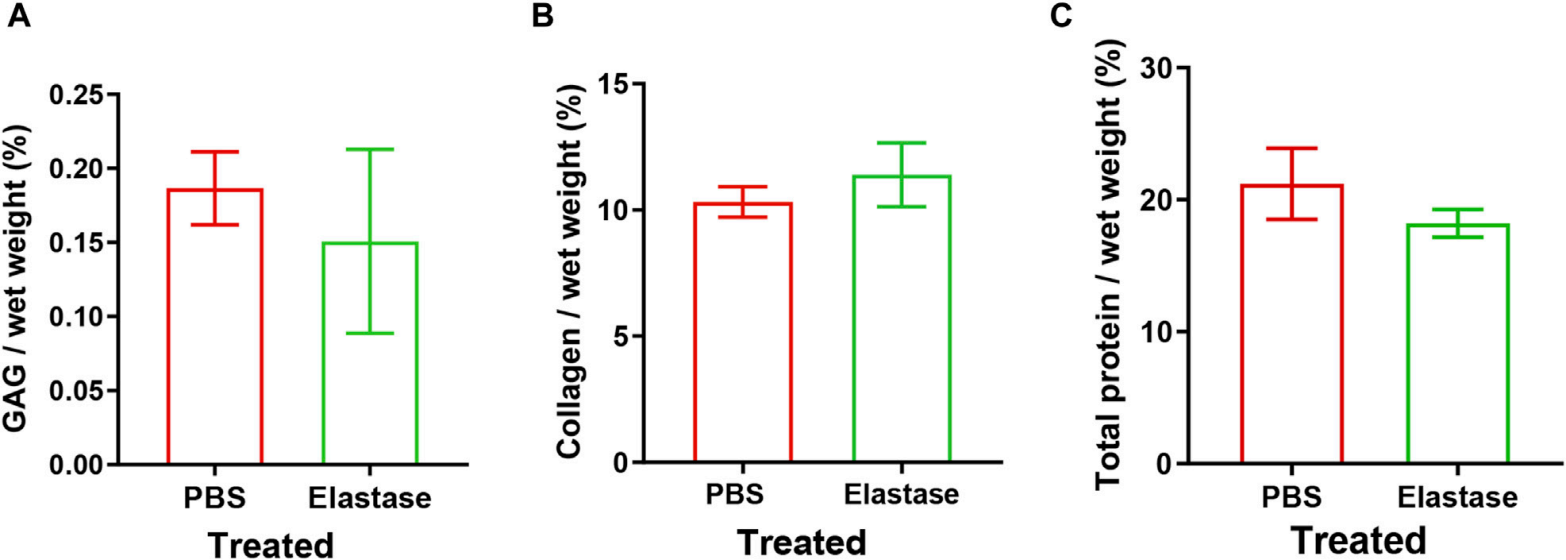
Biochemical indexes of patellar tendon incubated with 5 U/mL elastase or PBS solution. PBS is phosphate-buffered saline. **(A–C)** The content of sGAG, hydroxyproline, and total protein remaining after PBS or elastase treatment, respectively.

Compared with the PBS control group, the GAGs and total protein contents in the enzyme treatment group decreased; but no significant effect was observed (Figures 3A, C). Similarly, elastase treatment insignificantly affected collagen (Figure 3B). Therefore, it was shown that incubation with elastase had no significant effect on other biochemical indices possibly related to mechanics.

### 3.3 Elastin degradation significantly affected the tensile mechanical properties of patellar tendon

**FIGURE 4:**
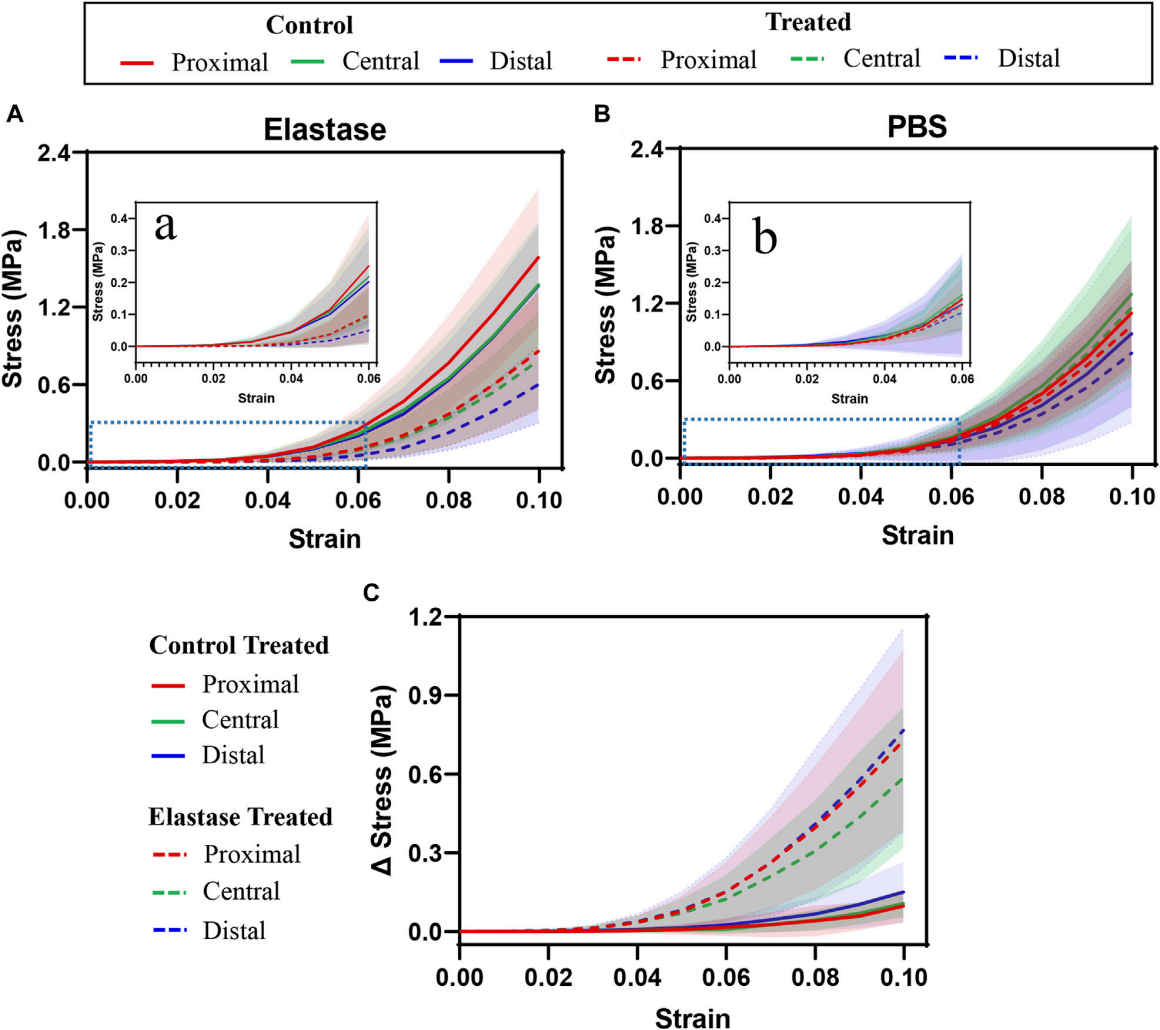
Tensile stress-strain curves of three different regions of patellar tendon treated with PBS or elastase. The control group contained the experimental data of each group before treatment. PBS is phosphate-buffered saline. **(A,B)** Mechanical curves of 5 U/mL elastase or PBS solution before and after incubation are shown. **(C)** Difference before and after treatment with PBS or 5 U/mL elastase. **(A,B)** Enlarged view of the blue dotted line in Figure **(A)** and **(B)**. Shaded area: standard deviation.

The stress at the proximal patellar tendon before treatment was greater than that at the central and distal tendons; however, the central and distal patellar tendons coincided (Figure 4A). The results in Figure 4B differed from those in Figure 4A, and the mechanics of the patellar tendon area were that the central was greater than the proximal, and the proximal was greater than the distal. In all three regions, the stress before elastase treatment was not statistically different from that before PBS treatment (*p* > 0.05). In the patellar tendon regions, the stress after treatment was appeared to be lower than that before treatment (elastase group: proximal *p* < 0.002, central *p* < 0.027, and distal *p* < 0.08; PBS group: proximal *p* < 0.281, central *p* < 0.055, and distal *p* < 0.009) (Figures 4A, B); however, compared with the PBS control group, the stress of the patellar tendon after elastase incubation was significantly reduced (proximal *p* < 0.004, central *p* < 0.003, and distal *p* < 0.004) (Figure 4C). The toe area of the patellar tendon after elastase treatment was longer than that before treatment, and it was stressed with an increase in fixture displacement after 4% strain (Figure 4A). However, this trend was not observed in the PBS treatment group; the toe region was similar before and after treatment, and the tissue was stressed at 4% (Figure 4B).

**FIGURE 5:**
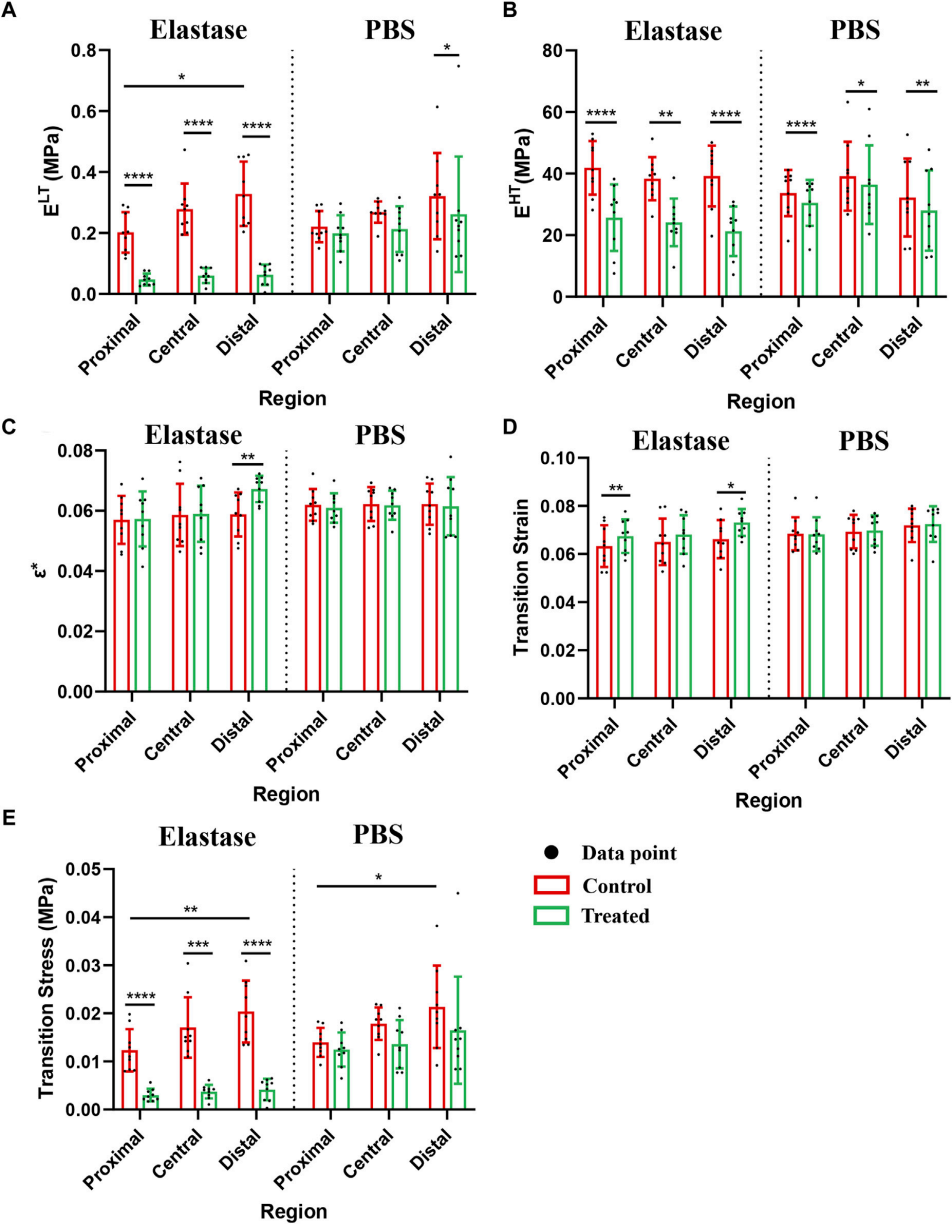
Mechanical properties of patellar tendon before and after treatment with three different regions. The control group contained the experimental data of each group before treatment. PBS is phosphate-buffered saline. **(A**–**E)** Comparison of the low-tension modulus, high-tension modulus, index of extensibility, transition strain, and transition stress before and after treatment with 5 U/mL elastase or PBS, respectively. * for *p* < 0.05, ** for *p* < 0.01, *** for *p* < 0.001, **** for *p* < 0.0001.

Although the moduli of the patellar tendon regions differed before treatment, these differences were statistically insignificant. The low tensile modulus of the distal patellar tendon before treatment was significantly higher than that of the proximal patellar tendon (*p* = 0.015) (Figure 5A). After elastin degradation in the three patellar tendon regions, the low tensile modulus decreased significantly (75%, 77%, and 80% in the proximal, central, and distal, respectively; all *p* < 0.0001) (Figure 5A), whereas the high tensile modulus decreased significantly (40%, 38%, and 47% in the proximal, central, and distal, respectively; *p* < 0.0001, *p* = 0.0039, *p* < 0.0001, respectively) (Figure 5B), and the stiffness of the tissue decreased. Although a trend of tissue stiffness reduction was found in the PBS group, it was insignificant in all regions, and the reduction was smaller than that in the enzyme group (Figures 5A, B). In addition, the ductility index among the regions before treatment differed insignificantly. Only the distal patellar tendon significantly increased in ductility index (by 16%) after enzymatic digestion of elastin (*p* = 0.0039) (Figure 5C).

After elastase treatment, the transition strain increased, particularly in the proximal and distal patellar tendons (*p* = 0.0039, *p* = 0.0359, respectively, Figure 5 D). The transition strain between the patellar tendon regions differed insignificantly; however, the transition stress at the distal patellar tendon was greater than that at the proximal region (*p* = 0.0207, *p* = 0.0293) (Figure 5E). Similarly, the transition stress decreased significantly after elastin degradation in different patellar tendon regions (75%, 77%, and 79% in the proximal, central, and distal, respectively; *p* < 0.0001, *p* = 0.0001, *p* < 0.0001, respectively) (Figure 5E). Although a downward trend was observed in the PBS treatment group, this difference was statistically insignificant (Figure 5E).

### 3.4 Effect of elastin degradation on stress relaxation mechanical properties of the patellar tendon

**FIGURE 6:**
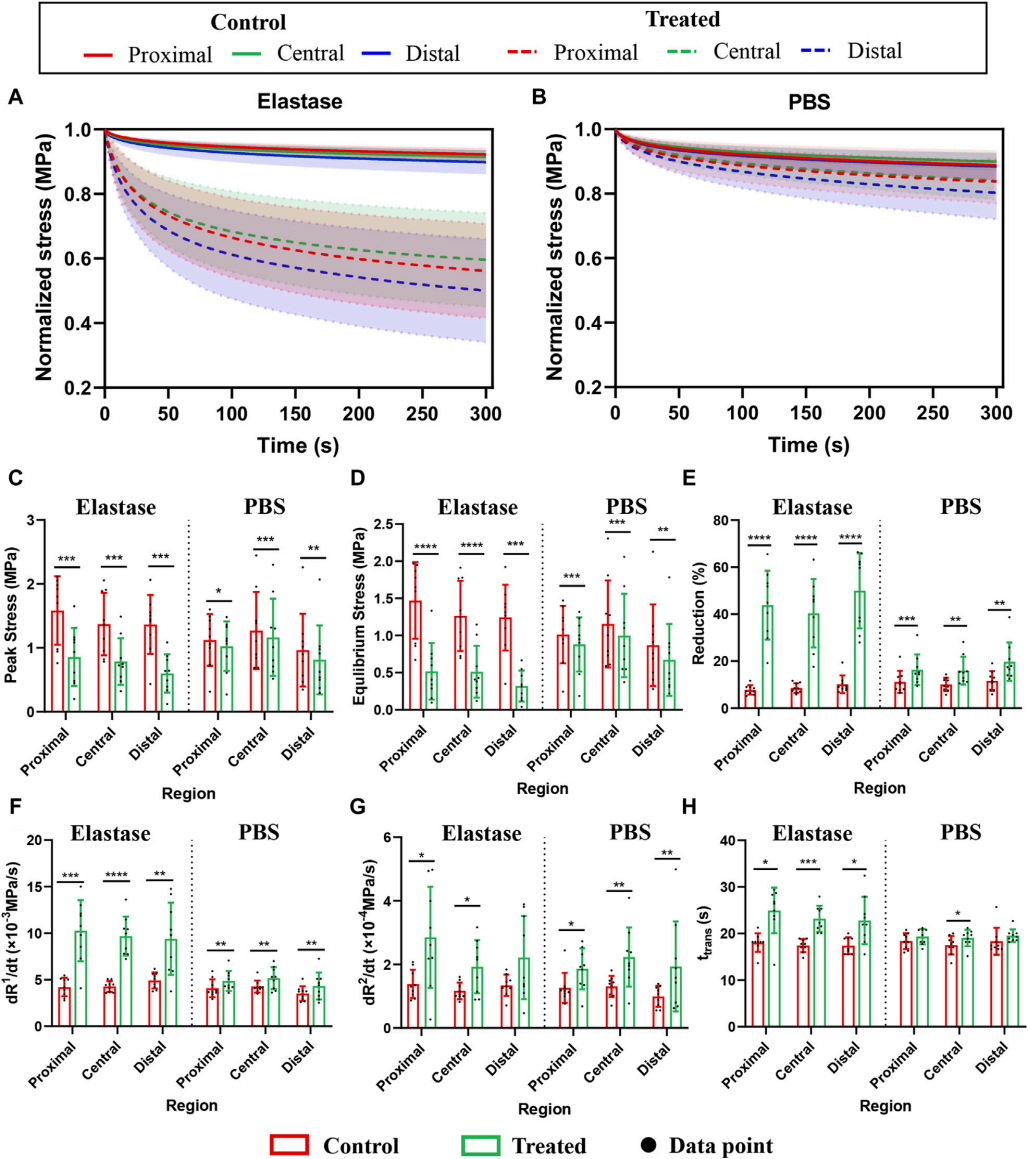
Stress relaxation properties of three different regions of the patellar tendon before and after treatment. The control group contained the experimental data of each group before treatment. PBS is phosphate-buffered saline. **(A)** Normalized stress-time curves before and after incubation with 5 U/mL elastase. **(B)** Normalized stress-time curves before and after PBS treatment. **(C–H)** Comparison of peak stress, equilibrium stress, relaxation percentage, initial slope, saturation slope, and transition time before and after treatment with 5 U/mL elastase or PBS, respectively. * for *p* < 0.05, ** for *p* < 0.01, *** for *p* < 0.001, **** for *p* < 0.0001.

Significant stress relaxation was observed in all patellar tendon regions, and they had similar stress (Figure 6). Compared with the PBS control group, elastin showed a more evident downward trend after degradation, and the stress reduction value was concurrently greater (Figures 6A, B).

The quantitative performance of stress relaxation in the patellar tendon regions before treatment differed insignificantly; however, after enzyme treatment, the peak stress was significantly reduced (47%, 43%, and 56% in the proximal, central, and distal, respectively; *p* = 0.0002, *p* = 0.0002, *p* = 0.0004, respectively) (Figure 6C), and the equilibrium stress was also significantly reduced (66%, 62%, and 74% in the proximal, central, and distal, respectively; *p* < 0.0001, *p* < 0.0001, *p* = 0.0001, respectively) (Figure 6D). However, compared with the PBS group, the range of stress reduction after elastin degradation was larger (Figures 6C, D). The relaxation percentage of the enzyme treatment group reached 40% in the three regions, higher than that of the PBS group (Figure 6E).

Furthermore, the initial slope, saturation slope, or transition time among the three regions of the patellar tendon regions differed insignificantly (Figures 6F–H). The patellar tendons in different regions showed an increasing trend after treatment in the PBS control and enzyme treatment groups. In the enzyme treatment group, the initial slope increased significantly (59%, 56%, and 48% in the proximal, central, and distal, respectively; *p* = 0.0002, *p* < 0.0001, *p* = 0.0044, respectively) (Figure 6F), the saturation slope increased significantly (51%, 40%, and 39% in the proximal, central, and distal, respectively; *p* = 0.0159 *p* = 0.0106, *p* = 0.0572, respectively) (Figure 6G), and the transition time was also increased significantly (28%, 25%, and 24% in the proximal, central, and distal, respectively; *p* = 0.0126, *p* = 0.0004, *p* = 0.0186, respectively) (Figure 6H). But the changes of the three mechanical indexes in PBS treatment group were small (Figures 6F–H). As the differences among the patellar tendon regions were insignificant, the data before and after processing of the three regions were summed, and the average value was calculated. The initial slopes of the enzyme group before and after the control: 4.454 ± 0.81 and −9.79 ± 3.08, respectively; The saturation slopes: 1.297 ± 0.35 and −2.332 ± 1.24, respectively; The transition time: 17.62 ± 1.727 and 23.65 ± 4.253, respectively.

### 3.5 Constitutive modeling

#### 3.5.1 Hyperelastic constitutive model under uniaxial tensile

In this study, Yeoh’s hyperelastic constitutive model was used to fit the collected stress-strain data. The goodness-of-fit *R* ^2^ and NRMSE (Ngwangwa et al., 2022) showed an ideal fitting effect (Supplementary Figures S3A, B). The *R* ^2^ values were above 0.99 and NRMSE was less than 0.10, indicating that the fitting effect was significant. Based on this constitutive model, the stiffness parameters C_10_, C_20_, C_30_ were calculated before and after elastin degradation in the three patellar tendon regions (Table 1 and 2). In different regions, elastin degradation greatly reduced the stiffness of the material. However, the stiffness of the material in the PBS group fluctuated slightly after treatment.

**TABLE 1:** Changes of tensile fitting parameters of patellar tendon before or after 5 U/mL elastase treatment.

	Control					Elastase treated				
Region	C_10_ (MPa)	C_20_ (MPa)	C_30_ (MPa)	*R* ^2^	NRMSE	C_10_ (MPa)	C_20_ (MPa)	C_30_ (MPa)	*R* ^2^	NRMSE
Proximal	−0.23 ± 0.20	44.87 ± 40.01	290.10 ± 563.88	0.99	0.05	−0.13 ± 0.16	16.13 ± 23.30	358.51 ± 326.99	0.99	0.06
Central	−0.14 ± 0.21	34.66 ± 39.49	314.03 ± 527.61	0.99	0.04	−0.11 ± 0.12	14.61 ± 21.37	319.26 ± 293.00	0.99	0.07
Distal	−0.13 ± 0.17	30.49 ± 32.06	412.80 ± 494.13	0.99	0.05	−0.04 ± 0.07	3.32 ± 8.75	417.95 ± 191.58	0.99	0.09

**TABLE 2:** Changes of tensile fitting parameters of patellar tendon before or after PBS treatment.

	Control					PBS treated				
Region	C_10_ (MPa)	C_20_ (MPa)	C_30_ (MPa)	*R* ^2^	NRMSE	C_10_ (MPa)	C_20_ (MPa)	C_30_ (MPa)	*R* ^2^	NRMSE
Proximal	−0.13 ± 0.14	22.18 ± 22.80	414.71 ± 274.09	0.99	0.05	−0.13 ± 0.13	20.90 ± 19.88	374.23 ± 219.02	0.99	0.06
Central	−0.11 ± 0.23	22.39 ± 31.41	522.74 ± 232.99	0.99	0.04	−0.12 ± 0.18	21.65 ± 27.97	460.31 ± 231.87	0.99	0.05
Distal	−0.00 ± 0.09	11.89 ± 31.59	473.75 ± 427.80	0.99	0.05	−0.02 ± 0.08	10.09 ± 28.81	406.65 ± 409.71	0.99	0.05

#### 3.5.2 Viscoelastic constitutive model under stress relaxation

The Prony series were used to fit the stress-relaxation data of the patellar tendon. It showed a better fitting effect of the experimental data (Supplementary Figures S3C, D). The goodness-of-fit *R* ^2^ and NRMSE were also used to measure the fitting effect, where the data *R* ^2^ values were above 0.99 and NRMSE was close to 0, indicating that the fitting effect was significant. The fitting parameters before and after elastin degradation in the three different areas of the patellar tendon were calculated (Table 3 and 4). In different regions, it was found that the instantaneous modulus of elastin decreased after degradation, but the material parameters ɑ increased, τ_1_ increased, but τ_2_ decreased. In the PBS control group, the instantaneous modulus decreased, and the material parameters ɑ increased, with a range smaller than that in the enzyme treatment group, and τ increased.

**TABLE 3:** Fitting parameters of second-order Prony series before or after 5 U/mL elastase treatment of the patellar tendon.

	Control							Elastase treated						
Region	G_0_ (MPa)	ɑ_1_ (MPa)	τ_1_ (s)	ɑ_2_ (MPa)	τ_2_ (s)	*R* ^2^	NRMSE	G_0_ (MPa)	ɑ_1_ (MPa)	τ_1_ (s)	ɑ_2_ (MPa)	τ_2_ (s)	*R* ^2^	NRMSE
Proximal	5.28 ± 1.78	0.03 ± 0.01	14.21 ± 1.11	0.06 ± 0.02	195.31 ± 18.18	0.99	0.00	2.85 ± 1.52	0.19 ± 0.09	16.57 ± 4.54	0.28 ± 0.10	146.11 ± 39.61	0.99	0.00
Central	4.57 ± 1.63	0.03 ± 0.01	13.44 ± 1.49	0.06 ± 0.01	181.87 ± 38.91	0.99	0.00	2.61 ± 1.21	0.18 ± 0.08	15.57 ± 2.63	0.24 ± 0.08	135.98 ± 26.03	0.99	0.00
Distal	4.55 ± 1.54	0.03 ± 0.01	12.59 ± 1.71	0.07 ± 0.03	167.86 ± 13.88	0.99	0.00	1.98 ± 0.99	0.24 ± 0.12	16.41 ± 4.41	0.32 ± 0.11	204.17 ± 163.53	0.99	0.00

**TABLE 4:** Fitting parameters of second-order Prony series before or after PBS treatment of the patellar tendon.

	Control							Elastase treated						
Region	G_0_ (MPa)	ɑ_1_ (MPa)	τ_1_ (s)	ɑ_2_ (MPa)	τ_2_ (s)	*R* ^2^	NRMSE	G_0_ (MPa)	ɑ_1_ (MPa)	τ_1_ (s)	ɑ_2_ (MPa)	τ_2_ (s)	*R* ^2^	NRMSE
Proximal	3.74 ± 1.35	0.04 ± 0.02	13.47 ± 1.53	0.09 ± 0.03	188.08 ± 18.60	0.99	0.00	3.41 ± 1.30	0.06 ± 0.03	14.82 ± 1.93	0.14 ± 0.05	218.25 ± 63.48	0.99	0.00
Central	4.22 ± 2.02	0.03 ± 0.01	12.94 ± 2.19	0.08 ± 0.03	186.47 ± 30.42	0.99	0.00	3.86 ± 2.01	0.05 ± 0.02	15.49 ± 1.98	0.15 ± 0.07	236.35 ± 58.32	0.99	0.00
Distal	3.21 ± 1.89	0.04 ± 0.02	13.88 ± 1.84	0.09 ± 0.03	188.37 ± 28.07	0.99	0.00	2.79 ± 1.79	0.06 ± 0.02	14.94 ± 1.09	0.18 ± 0.09	224.20 ± 72.87	0.99	0.00

### 3.6 Histological analysis

**FIGURE 7:**
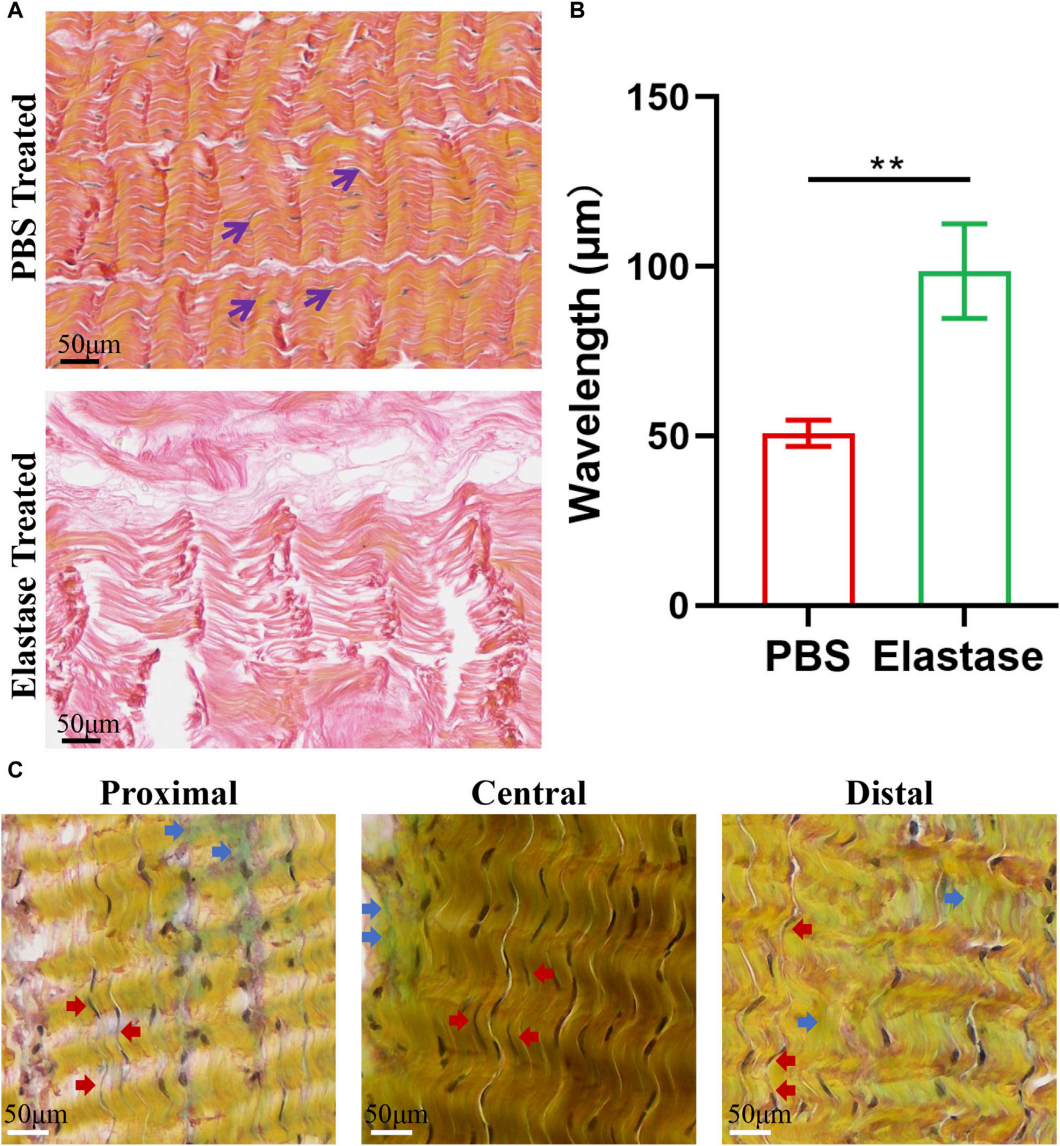
Typical histological staining of porcine patellar tendon. PBS is phosphate-buffered saline. **(A)** The structural diagrams of PBS treatment or 5 U/mL elastase treatment in VVG staining. After elastase treatment, the black elastic fibers disappeared, and the red collagen fibers became longer. **(B)** The wavelength of patellar tendon fibers treated with elastase increased. Black arrows denote elastic fibers. **(C)** Movat’s staining structure diagram of three regions of patellar tendon. Blue arrows indicated blue-green proteoglycans, and red arrows indicated black elastic fibers. Similar composition structures were observed in three regions, and black elastic fibers could be seen to be arranged along yellow collagen fibers in each region.

The patellar tendon was evaluated using VVG staining, and the black, slender elastic fibers in the patellar tendon were closely arranged along the red collagen fibers (Figure 7A). After incubation with elastase, elastin was degraded, inducing morphological changes in the collagen fibers (Figure 7A). The tissue became loose, and the wavelength of the collagen curl increased, which caused fiber straightening (Figure 7B).

Histological evaluation of Movat’s staining revealed that the three patellar tendon regions had similar microstructures, all comprising collagen fibers, cells, proteoglycans, and elastin fibers, and the inter bundle matrices between the collagen fiber bundles were evident (Figure 7C). Similarly, slender black elastin fibers could also be found in all patellar tendon areas, which were closely arranged along yellow collagen fibers.

### 3.7 SHG imaging of patellar tendon

**FIGURE 8:**
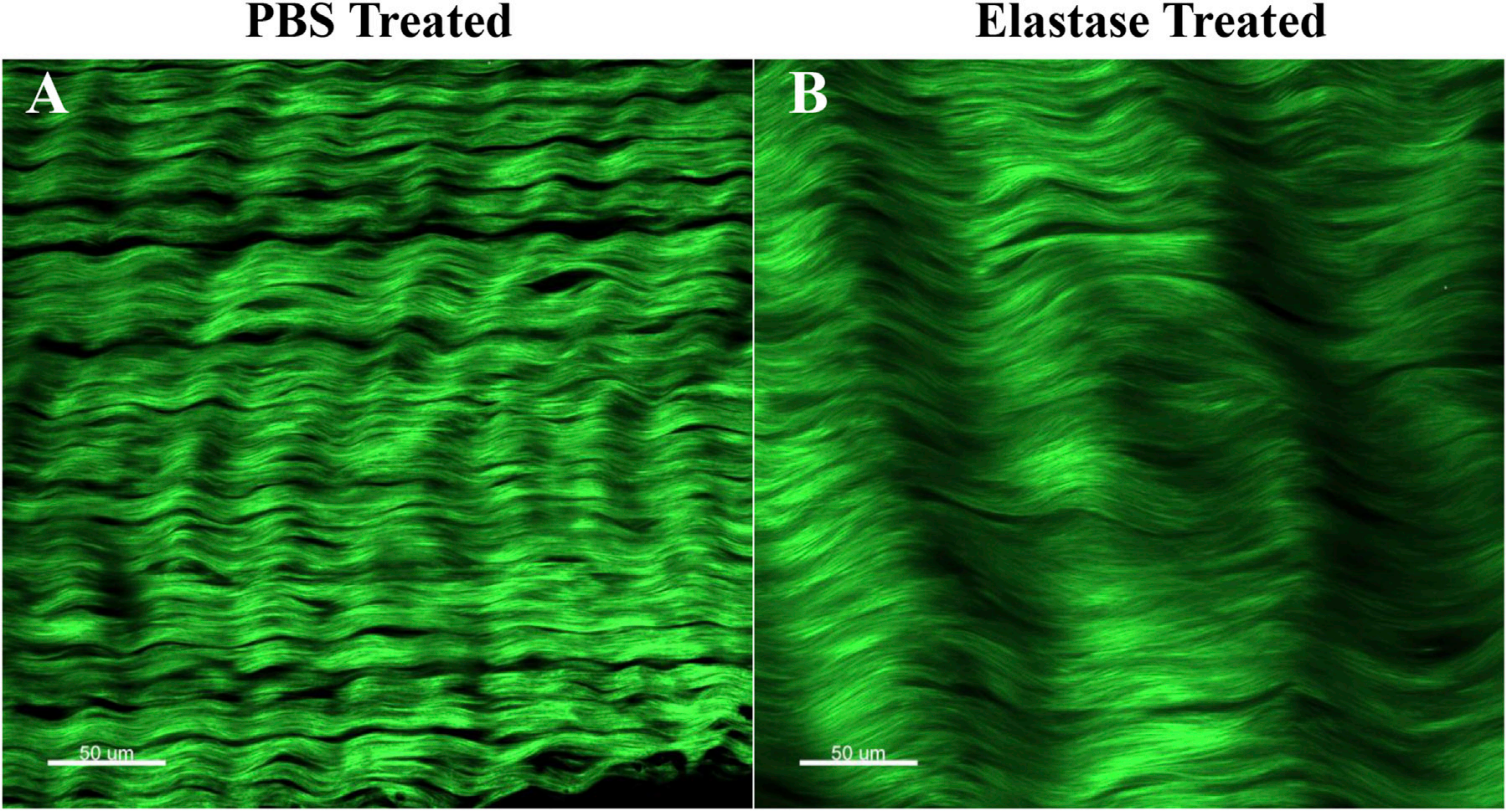
Two-photon imaging of patellar tendon. Green represented collagen fibers. PBS is phosphate-buffered saline. **(A)** Representative image after PBS treatment. **(B)** Representative image after elastase treatment.

The two-photon image of patellar tendon showed the characteristic structure of collagen fibers treated with PBS or elastase (Figure 8). Samples were imaged directly without special liquid fixation, and collagen fibers were displayed in green. In the samples before PBS or enzyme treatment, it was found that collagen fibers had periodic curl and were closely recruited by slender fiber bundles (Supplementary Figures S4A, B). However, compared with the structure before treatment, the collagen fibers in PBS control group remained tightly packed, and the integrity of collagen fibers was not damaged by elastin degradation (Figure 8). It was generally found that the degradation of elastin made collagen fibers looser and the distance between fibers relatively longer, which led to that the enzyme treatment group consisted of fewer fibers than the PBS group in the same size images.

## 4 Discussion

In this study, biomechanics and optical imaging were combined to explore the mechanical properties and structural changes of the same tissue sample in three different regions of patellar tendon before and after elastin degradation, which evaluated the contribution of elastin to the mechanical integrity and fiber structure arrangement of patellar tendon. The Movat’s staining results of the three regions suggest that their mechanical properties differed insignificantly, possibly owing to their similar structures. Mechanical characterization showed that the degradation of elastin reduced tensile stress, modulus and transition strain. In the stress relaxation experiment, the influence of elastin on the viscoelastic behavior of the patellar tendon was better understood. After elastin degradation, the initial and saturation slopes increased, the transition time became longer, the relaxation percentage increased, and the stress reduction amplitude increased. Optical imaging results showed that elastin degradation reduced the degree of fiber recruitment of patellar tendon. These results aid in understanding the relationship between patellar tendon diseases, microstructure, and mechanics.

Two-photon imaging and histological staining revealed the spatial arrangement of elastin and collagen fibers in patellar tendon. The decrease of collagen fiber fluctuation caused by elastin degradation could lead to mechanical changes, which better explained the mechanical contribution of fiber structure to the patellar tendon. Soft tissue shows a typical nonlinear stress-strain curve under a tensile load, which has four typical regions: low stress-strain toe linear elastic state, highly nonlinear transition state, linear elastic state, and finally, yield and failure of the structure (Herbert et al., 2016; Li et al., 2019). This study highlighted the contribution of elastin to the biomechanical nonlinear mechanical behavior of patellar tendon. After enzyme treatment, the low stress-strain region of the patellar tendon was prolonged, stress exertion began after 4% strain, and the transition strain and ductility index increased, indicating that elastin degradation significantly affected the mechanical properties of the toe region. When performing two-photon imaging, the clamp distance before or after sample treatment was the same, and the phenomenon found in the imaging process could be considered as the effect of elastase treatment. This imaging method was similar to the mechanical test of samples, that is, the initial length was consistent before and after treatment. After enzyme treatment, the fiber arrangement of the sample was loose and the curling distance was prolonged, which led to that the treated sample had no stress at the same initial length, thus explaining the lengthening of the toe region in the nonlinear region. The morphological results of patellar tendon showed that there was a certain interaction between elastin and collagen fiber. The interaction between them was destroyed, which caused the damage of mechanical properties, and might indicated that elastin played a bearing role in toe region (Chow et al., 2013). It is said that insufficient elastin changes the recruitment of collagen fibers, causing tissue elongation, and the straightness of collagen fibers leads to a delay in the ability of tissues to bear loads (Grant et al., 2015; Fazaeli et al., 2020). After elastin degradation, the patellar tendon is in an extended state without a preload, similar to the phenomenon where the joint relaxes (Eekhoff et al., 2023), and the natural tissue becomes more malleable, severely harming the human body. In this study, the low and high moduli after enzyme treatment were significantly reduced, which was similar to a study on bronchus (Mariano et al., 2023). It emphasizes the contribution of elastin to the low-stress mechanical behavior and tissue elasticity. The decrease in the high tensile modulus may be caused by the failure of collagen fibers to fully dominate the load owing to tissue elongation.

The patellar tendon is a viscoelastic material, and stress exhibits time-dependent behavior, causing a small amount of energy to dissipate, and the change in viscoelasticity affects the progress of the disease (Chaudhuri et al., 2020). In a stress relaxation experiment of elastin degradation, enzyme treatment significantly affected viscoelastic properties, and the percentage of total stress attenuation decreased significantly, providing experimental information for understanding how patellar tendons are affected by extracellular matrix composition changes and time-dependent behavior (Ross et al., 2021). The increase in the initial slope showed that tissue stress decreased faster after enzyme treatment, and the increase in saturation slope showed that the ability of tissue lacking elastin to reach an equilibrium state was relatively reduced; the increase in transition time representing relaxed shape also showed this point. These results indicate that microstructural elastin affects the viscoelastic properties of the patellar tendon, and changes in its content affect tissue homeostasis (Huang et al., 2019). The exact origin of the viscoelastic behavior of the tendon is unclear; however, stress relaxation results from the interaction between the matrix and fluid, which changes with time in the material (Screen et al., 2013). It is believed that stress relaxation may be caused by the relaxation of collagen fibers (Maritz et al., 2021) and that the degradation of elastin causes an arrangement change in the fiber structure, inducing a change in the stress relaxation phenomenon. This possibility is consistent with the histological results found in this study. Stress relaxation mechanisms can even affect tissue morphology and tumorigenesis (Elosegui-Artola et al., 2023). Viscoelasticity is an essential parameter in the design of tissue engineering and regenerative medicine materials, and its characteristics significantly influence cell behavior (Obuchowicz et al., 2019; Freedman et al., 2022b). Elastin should be added in the future manufacturing of biomaterials to make them more in line with the natural tissue structure and mechanical environment (Schmelzer et al., 2020).

Understanding the biomechanical characteristics of soft tissue is very important for the development of calculation model, which can provide more physical insights (Jan et al., 2022). Soft tissue is a nonlinear, heterogeneous and anisotropic material, but there is no unified constitutive model to describe the properties of the material in previous studies. For example, the compressive Neo-Hookean strain-energy was used to describe the mechanical response of bronchi (Eskandari et al., 2019). Study on the effect of GAGs on the recruitment of aortic collagen fibers based on structural-based constitutive model (Mattson et al., 2019). A new strain energy function based on the geometric arrangement of fibrils and Holzapfel-Gasser-Ogden model were used to compare and analyze the mechanical behavior of ligaments and tendons (Shearer, 2015). At the same time, many studies assume that soft tissue is an incompressible hyperelastic and isotropic material, and use Ogden model, Yeoh model or other models to describe the stress-strain relationship of skin and tendon structure (Cheng and Gan, 2008; Remache et al., 2018). Compared with previous studies, this study adopted a simplified Yeoh model, which was proved to be the most suitable for fitting hyperelastic and transversely isotropic tendons (Liber-Kneć and Łagan, 2020). In the existing research, there are few studies on directly obtaining the mechanical data of tendon elastin before and after degradation and fitting the constitutive model at the same time. The constitutive model of this study was established in an ideal state, and it was fitted by Yeoh model, a hyperelastic constitutive model. *R* ^2^ and NRMSE showed that the fitting effect of this model was great and suitable for this study. It deduced the material parameters of the constitutive model of the patellar tendon before and after elastin degradation. Similarly, elastin degradation reportedly decreases material stiffness. However, some models based on structure (distribution of fiber networks) can also be used to predict soft tissue responses (Henninger et al., 2019). Soft tissue is a viscoelastic material. Many studies have also proposed a viscoelastic model to simulate stress relaxation behavior, such as quasi-linear viscoelastic (QLV) model (Duenwald et al., 2010). Since the theory of this model is linear viscosity hypothesis, it cannot fully describe the nonlinear viscoelastic behavior of various biological soft tissues (Shetye et al., 2014). However, Prony series is widely used and proved to be a constitutive equation that can effectively express the viscoelasticity of materials (Grega et al., 2020; Shearer et al., 2020; Li et al., 2021; Morrison et al., 2023). In this study, *R* ^2^ and NRMSE obtained by fitting the experimental data of stress relaxation of patellar tendon before and after treatment showed that Prony series had a good fitting effect and was suitable for this study. In the material parameters of the viscoelastic constitutive model of the three patellar tendon regions before and after elastin degradation, it was found that after enzyme treatment, the patellar tendon degraded by elastase exhibited greater stress attenuation and a longer relaxation time. Compared with the untreated tissue, elastin degradation increased the material parameter ɑ, indicating that the lack of elastin causes the patellar tendon to exhibit elastic behavior (Ross et al., 2021). The mechanical constitutive models established in this study are helpful to better understand the incidence and prevention of patellar tendon diseases, because they can simulate the biomechanical behavior of tissues and their components from the phenomenon (Khayyeri et al., 2016).

It showed that the stress of porcine ligament was reduced by shear test and transverse tension after elastase treatment (Henninger et al., 2015). The degradation of elastin leads to a significant decrease in the viscoelasticity of the interfascicular matrix of horse energy storage tendons, but it does not affect the fascicle mechanics (Godinho et al., 2021). However, the compression test of tendon after enzyme treatment was not found. It may be because tendons are more influenced by uniaxial direction (that is, fiber direction) *in vivo*, and axial tension is a conventional mechanical test method (Ristaniemi et al., 2021; Lake et al., 2023). Through uniaxial stretching, it is concluded that elastin has made a more significant contribution to the patellar tendon as an energy storage tendon. There may be some differences in the mechanical properties of different species and different types of tendons, which may be used to explain different research results. Previous studies have also used elastin knockout mice to reduce elastin content, and the results of this method are inconsistent with those of this study (Eekhoff et al., 2017). However, using elastase to break down elastin within the tissue is completely different from the elastin knockout mice. This inconsistency can be attributed to the differences in their development conditions. Elastin in transgenic mice does not develop normally, and there is a compensation mechanism, which may lead to the result that it is not entirely due to the decrease of elastin (Eekhoff et al., 2017; Eekhoff et al., 2021). Because of the lack of elastin, the structural relationship and interaction between elastin and collagen fiber cannot be determined. However, the patellar tendon of pigs treated with elastase developed normally, and the effect of selectively degrading elastin on the structure and function of normal tendon can be studied. This is more suitable for guiding the repair and reconstruction of patellar tendon injury and designing biomaterials.

During the evolution of tendon lesions, collagen fibers were found to relax and curl unevenly (Wu et al., 2020; Mohindra et al., 2022), which was consistent with the morphological changes in collagen fibers caused by elastin degradation in this study. It provided evidence for tendon lesions caused by elastin degradation. Without the protection of elastin, collagen fibers were easily damaged and needed to be repaired for a long time, and long-term accumulation would lead to tissue damage or fracture (Naya and Takanari, 2023). In tendon tissue engineering, replacement and regeneration are challenging because the scaffold must ensure sufficient hierarchical structure and mechanical properties to bear the load. To guide cell proliferation and growth, the scaffold should provide a fiber network imitating the microstructural arrangement of collagen and elastic fibers in the extracellular matrix of the tendon (Bianchi et al., 2021). In addition, understanding the relationship between these microstructures and mechanics is helpful for tendon reconstruction so that the reconstructed graft can better fit the microstructure of the natural tendon structure (Smith et al., 2019). This study found that elastin plays an important role in the elasticity and viscoelasticity of patellar tendon, which was not discussed in depth in the previous biomechanical research of patellar tendon, and it also led to the lack of biomaterial properties at present. At present, there are collagen-based biomaterials (Yuan et al., 2021; Maeda, et al., 2022) and novel knitted scaffold made of microfiber/nanofiber core–sheath yarns (Cai, et al., 2020) used in tendon tissue engineering. However, these biomaterials cannot completely copy the structural and mechanical properties of the original tendon. Therefore, in order to design biomaterials that are more in line with natural tissues (such as artificial patellar tendon), elastin should be added. That is to say, when making different proportions of collagen-based biomaterials, different proportions of elastin should be added to make the mechanical properties of the materials better simulate the tissue, and these materials should have the spatial relationship between elastin and collagen fibers. The scaffold can be coated with elastin by electrospinning and bioprinting, and the hydrogel structure of elastin can be made.

This study has some limitations. First, the sample size is small in the study, although this number has been recognized by some studies (Fang and Lake, 2016; Fazaeli et al., 2020), it will also have some limitations on the results. Second, patellar tendon is an anisotropic material. The constitutive model established in this study assumed that it was transversely isotropic and divided the experimental data of tension and stress relaxation into two parts for fitting (Khayyeri et al., 2016; Remache et al., 2018). The transversely isotropic model cannot fully represent the real properties of materials. Future research should be devoted to establishing a constitutive model that conforms to the nonlinearity, anisotropy, and viscoelasticity of the patellar tendon and includes microstructures such as elastin and collagen fiber to obtain more real material parameters. Third, the samples of this study were from animals, and human samples should be selected to further verify the conclusions of this study so that the research results can be used in clinical practice. Fourth, only uniaxial tensile test was carried out in this study, but the mechanical environment of patellar tendon in the body is complex. Exploring the directional mechanical effect of elastase therapy can be an avenue for future studies. Finally, only the role of elastin was considered, we should consider the effect of collagen fiber and its degradation in the future. To further improve the patellar tendon model, it should be combined with imaging technology and materials science to further observe the connection components and their functions between elastin and collagen fibers in patellar tendon (Durgam et al., 2020; Sallehuddin et al., 2022).

In summary, this study provides an insight into the previously uninvestigated effects of elastin on the mechanical properties and fiber structure of different regions of patellar tendon. Our results showed that there was no evident regional mechanical difference in the patellar tendon under load. The specific degradation of patellar tendon elastin changed the structural arrangement of collagen fibers and affected the elastic mechanical behavior and viscoelastic properties of the tissues. Through imaging technology and biomechanical experiments, the microstructure-function relationship of patellar tendon was proved. The results benefit the model speculation of elastin fibers in normal, pathological, and injured connective tissues and guide the development of patellar tendon materials in tissue engineering and obtaining ideal properties.

## Data Availability

The datasets presented in this study can be found in online repositories. The names of the repository/repositories and accession number(s) can be found in the article/Supplementary Material.
