# Supplementary material for: The alteration of the structure and macroscopic mechanical response of porcine patellar tendon by elastase digestion
Source: Front Bioeng Biotechnol. 2024 Apr 17;12:1374352. doi: 10.3389/fbioe.2024.1374352 (PMC11061363; doi:10.3389/fbioe.2024.1374352)
Supplement: Supplementary file 1 [file DataSheet1.docx]

Supplementary Material

Effect of elastin degradation on the structure and mechanical response of porcine patellar tendon

*Xiaoyun Liu^1,2†^, Yuping Deng^1,2,3†^, Zeyu Liang^1†^, Dan Qiao^4†^, Wentian Zhang^1,5^, Mian Wang^1,2,6^, Feifei Li^1^, Jiannan Liu^1^, Yaobing Wu^1^, Guangxin Chen^7^, Yan Liu^8^, Wenchang Tan^2^, Jian Xing^7*^, Wenhua Huang^1,3,9*^,* *Dongliang Zhao^2*^, Yanbing Li^1*^*

*** Correspondence:** *Jian Xing:* [190423831@qq.com](mailto:190423831@qq.com); *Wenhua Huang:* Orthobiomech@163.com *; Dongliang Zhao:* [*zhaodongliang@szbl.ac.cn*](mailto:zhaodongliang@szbl.ac.cn)*; Yanbing Li:* [*hnlybup001@163.com*](mailto:hnlybup001@163.com)

# Supplementary Figures and Tables

## 1.1 Supplementary Figures


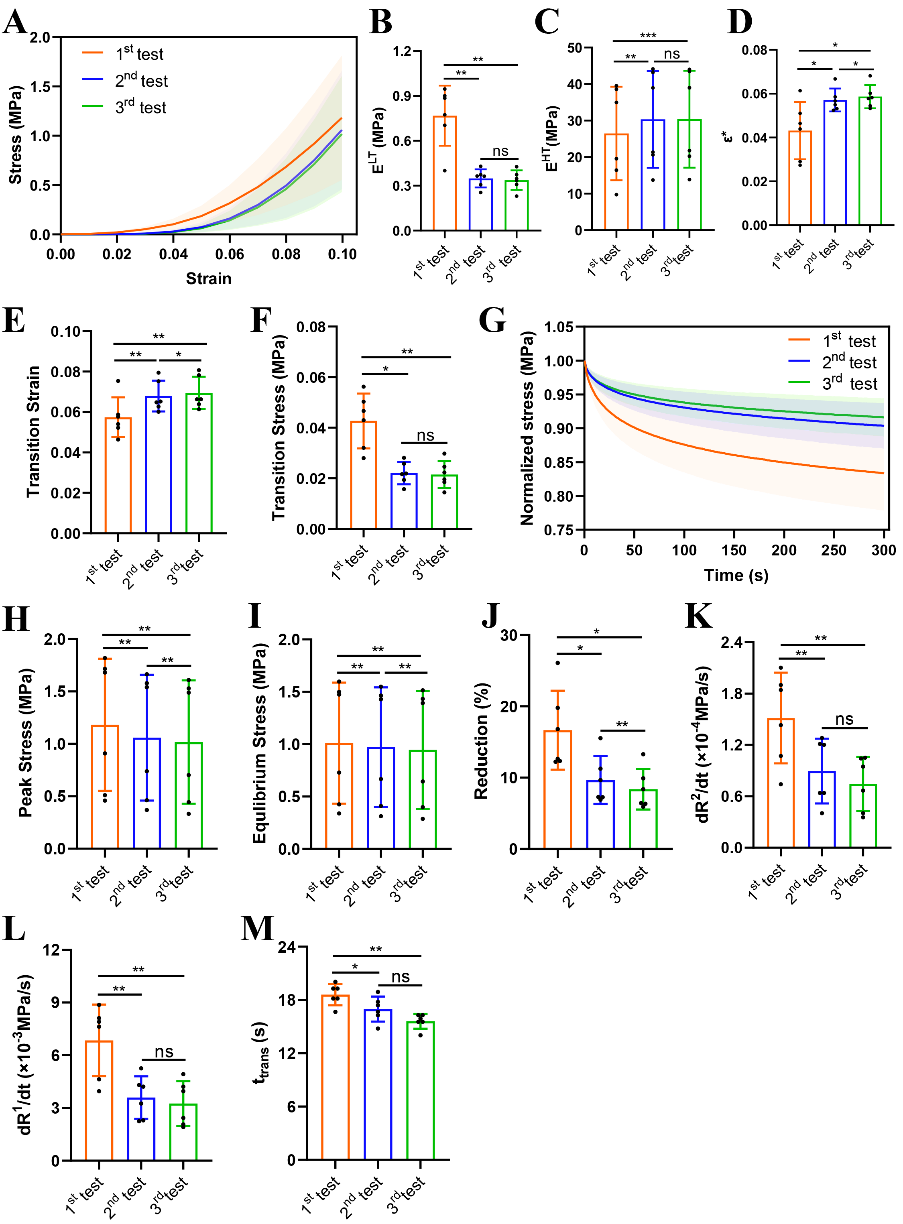


**Supplementary Figure 1.** Mechanical properties of three-time repeated stretching of patellar tendon (n=6). **(A)** Stress-strain curve of patellar tendon in three repeated tensile mechanical tests. Shaded areas: standard deviation range. (**B**-**F**) Comparison of low tensile modulus, high tensile modulus, ductility indexes, transition strain, and transition stress of three-time repeated tensile mechanical test. (**G**) Normalized stress-time curve of three repeated stress relaxation tests of patellar tendon. Shaded areas: standard deviation range. (**H**-**M**) Comparison of peak stress, equilibrium stress, relaxation percentage, initial slopes, saturation slopes, and transition time of three-time repeated tensile mechanical test. * for *p*<0.05, ** for *p*<0.01, *** for *p*<0.001, ns was not statistically significant.


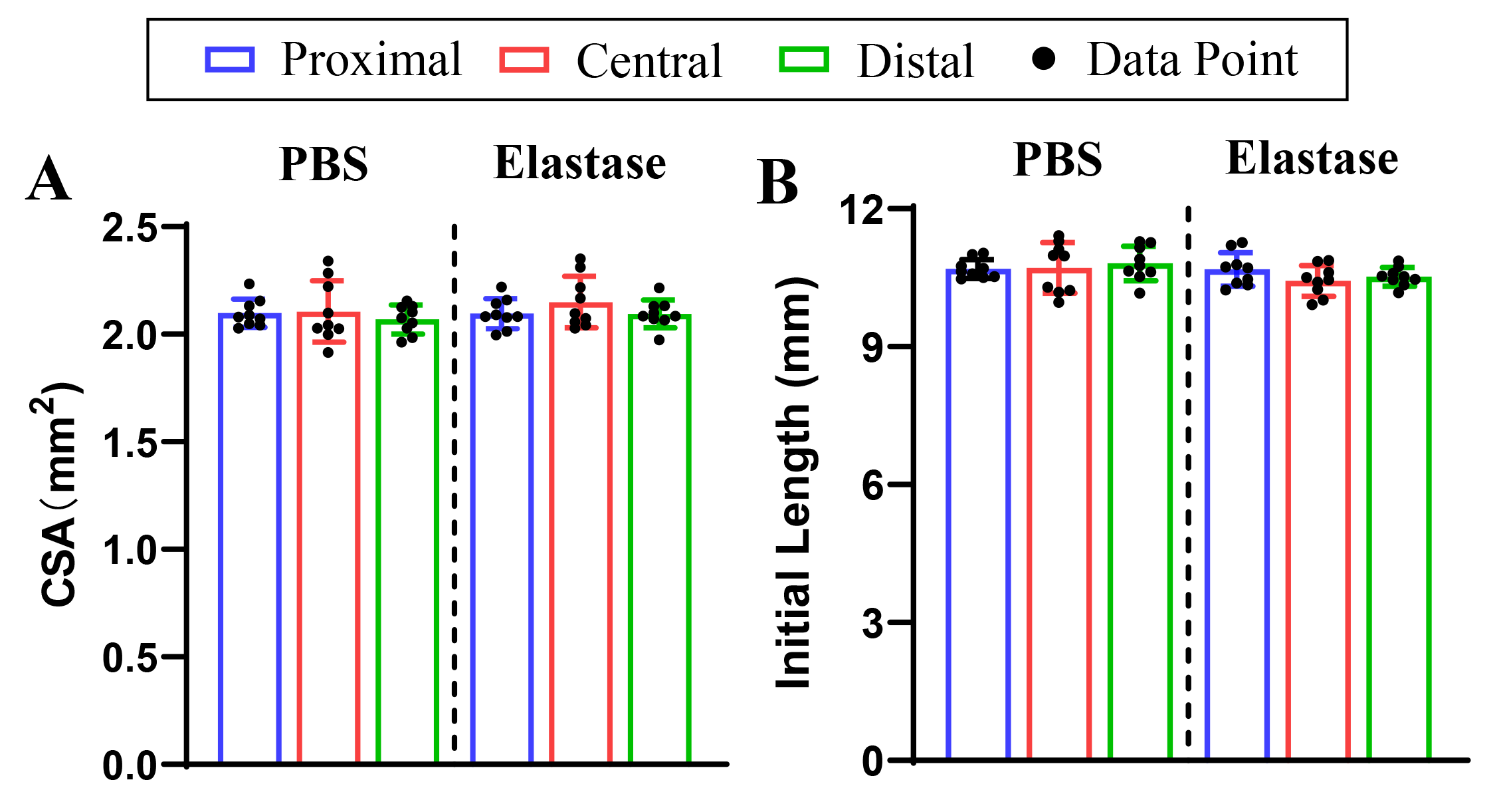


**Supplementary Figure 2.** Basic data of porcine patellar tendon specimen. (**A**) Cross-sectional area of PBS or enzyme treatment group. (**B**) Initial length of PBS or enzyme treatment group.


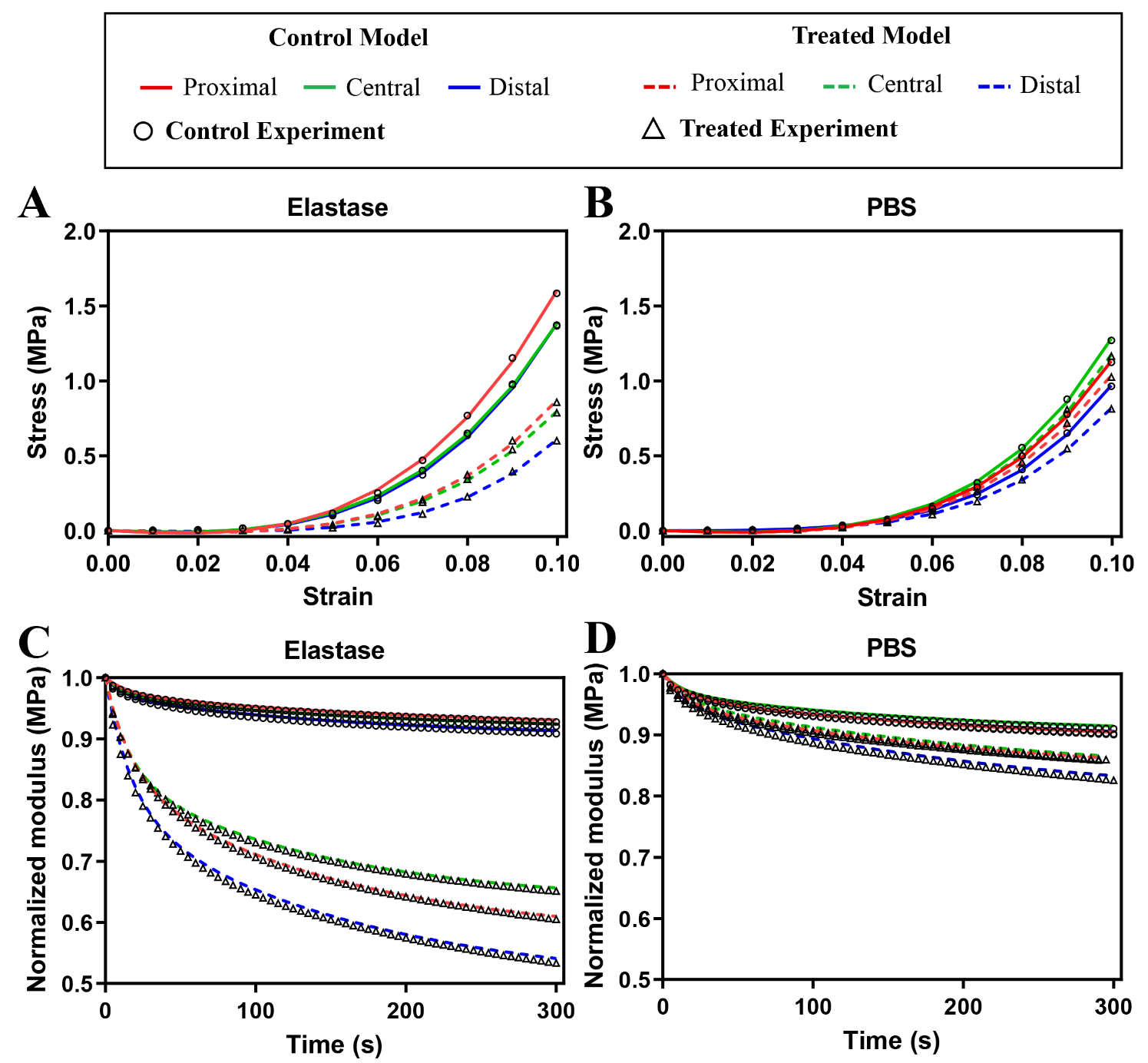


**Supplementary Figure 3.** Fitting results of three different regions of patellar tendon treated by PBS or elastase (n=9). Control was the experimental data before treatment in each group, and the good fitting result of the constitutive model was shown in the figure. (**A**, **B**) The fitting curves of hyperelastic constitutive model before and after incubation with 5 U/mL elastase or PBS were respectively shown. (**C**, **D**) The fitting curves of modulus normalization before and after incubation with 5 U/mL elastase or PBS were respectively shown.


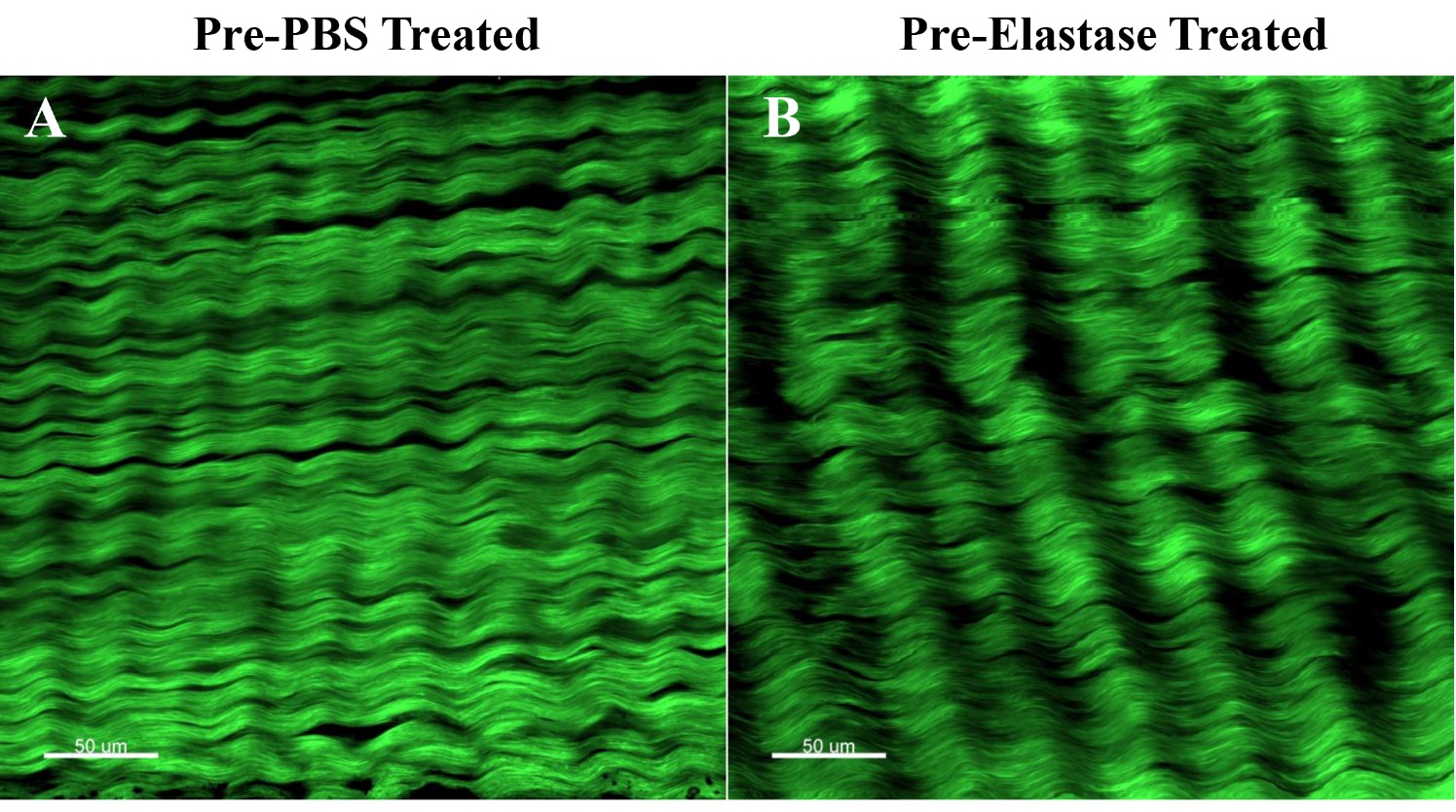


**Supplementary Figure 4.** Two-photon imaging of patellar tendon. Green represented collagen fibers. (**A**) Representative image before PBS treatment. (**B**) Representative image before elastase treatment.

## Supplementary Tables

**Supplementary Table 1. Detailed analysis of sample size.** **PBS is phosphate-buffered saline.**

| Experiment | | Sample Size | |
| --- | --- | --- | --- |
| Movat's staining | Proximal region | 1 | |
|  | Central region | 1 | |
|  | Distal region | 1 | |
|  | | **PBS group** | **Elastase group** |
| Biochemical Analysis | Collagen | 3 | 3 |
|  | GAGs | 3 | 3 |
|  | total protein | 3 | 3 |
| Mechanical Test | Proximal region | 9 | 9 |
|  | Central region | 9 | 9 |
|  | Distal region | 9 | 9 |
| Two-photon microscopy |  | 1 | 1 |
